# MSW-Mamba-Det: Multi-Scale Windowed State-Space Modeling for End-to-End Defect Detection in Photovoltaic Module Electroluminescence Images

**DOI:** 10.3390/s26092616

**Published:** 2026-04-23

**Authors:** Xiaofeng Wang, Haojie Hu, Xiao Hao, Weiguang Ma

**Affiliations:** 1 School of Electric Power, Civil Engineering and Architecture, Shanxi University, Taiyuan 030006, China; wangxiaofeng@sxu.edu.cn (X.W.); huhaojie@sxu.edu.cn (H.H.); 2 School of Physics and Electronic Engineering, Shanxi University, Taiyuan 030006, China; haoxiao@sxu.edu.cn; 3 State Key Laboratory of Quantum Optics Technologies and Devices, Institute of Laser Spectroscopy, Shanxi University, Taiyuan 030006, China; 4 Collaborative Innovation Center of Extreme Optics, Shanxi University, Taiyuan 030006, China

**Keywords:** electroluminescence imaging, photovoltaic modules, defect detection, state-space model, end-to-end detection

## Abstract

Electroluminescence (EL) imaging is widely used for photovoltaic (PV) module inspection, yet EL defect detection remains challenging due to the need for high-resolution inputs, low-contrast defects, and strong structured background patterns. To address these issues, we propose MSW-Mamba-Det, an end-to-end defect detection framework built on RT-DETR, comprising three components. (1) MSW-Mamba, a multi-scale windowed state-space module, adopts a Local/Stripe/Grid architecture to jointly model fine details and long-range dependencies; the Stripe branch strengthens directional continuity for elongated defects, while the Grid branch introduces coarse global context to improve cross-region consistency. Saliency- and gradient-guided gating is further used to suppress background-induced false responses. (2) DetailAware compensates for detail attenuation by restoring high-frequency textures and edges through multi-scale local enhancement, and applies pixel-wise adaptive gating to integrate global semantics and mitigate smoothing effects in deep representations. (3) PAFB (Pyramid Attention Fusion Block) aligns adjacent-scale features and improves multi-scale fusion, enhancing localization stability across defect sizes. Experiments on two public EL datasets show that MSW-Mamba-Det achieves $AP_{50:95}$ of 60.4% on PV-Multi-Defect-main and 68.0% on PVEL-AD, improving over RT-DETR by 2.5 points (from 57.9% to 60.4%) and 2.2 points (from 65.8% to 68.0%), respectively. MSW-Mamba-Det also outperforms 12 representative baselines, including CNN-, Transformer-, and recent YOLO-based models, in $AP_{50:95}$ on both datasets, with particularly strong performance on medium and large defects. These results demonstrate the effectiveness of the proposed modules for robust PV EL defect inspection under low-contrast and structured-background conditions.

## 1. Introduction

As the core energy-conversion unit of photovoltaic (PV) systems, accurate detection of cell-level defects in photovoltaic modules is essential to ensure stable operation and improve energy yield [1,2,3]. In this context, electroluminescence (EL) imaging provides a direct visualization of carrier recombination and current transport in PV cells under dark-field conditions [4]. Owing to its high sensitivity to early-stage defects—such as scratches, broken fingers, cold solder joints, and microcracks—EL-based inspection has become an important tool for both manufacturing quality control and in-field maintenance [5]. However, manual interpretation is time-consuming and subjective, so its accuracy often depends on the inspector’s experience [6]. This motivates the development of high-precision automated defect detection algorithms for PV EL images to enable reliable defect localization and identification.

Existing deep-learning-based methods for PV EL defect detection can be broadly grouped into two categories [7]. The first category includes anchor-based two-stage and single-stage CNN-based detectors that rely on hand-designed priors [8,9]. The second category includes end-to-end detectors based on Transformer-style set prediction [10].

In practice, PV EL images exhibit high resolution, weak contrast, and a strong structured background. Defects such as microcracks are often elongated and show low grayscale contrast [11]. Meanwhile, repetitive patterns induced by metal fingers, busbars, and (for polycrystalline modules) grain boundaries introduce prominent background interference [12]. Under these conditions, CNN backbones that mainly rely on local convolutions typically require deep stacking to enlarge the effective receptive field, which can limit their ability to model cross-region contextual correlations. As a result, they can produce missed detections or localization instability for elongated or weak-response defects [13]. In addition, anchor-based detectors require hand-designed priors (e.g., anchor scales and aspect ratios) and commonly use threshold-based non-maximum suppression (NMS) for post-processing [14]. Previous studies have shown that detection performance can be sensitive to anchor configurations. Fixed-threshold NMS can also suppress true positives in crowded or structurally repetitive regions, leading to missed detections [15,16]. Therefore, EL scenarios with strong repetitive structures often require careful prior design and post-processing tuning.

In contrast, DETR-style methods perform end-to-end detection via set prediction and Hungarian matching, thereby eliminating the need for anchors and NMS and simplifying the inference pipeline [17,18,19]. Nevertheless, applying dense self-attention to high-resolution EL images can incur substantial computational and memory costs due to the quadratic complexity of Transformer encoders [20]. Moreover, global feature aggregation may weaken local textures and edge cues, which can blur tiny defect boundaries and degrade localization stability [21]. Recent end-to-end detectors such as the Real-Time Detection Transformer (RT-DETR) improve multi-scale representations through intra-scale interaction and cross-scale fusion, achieving a better balance between accuracy and efficiency [22]. This makes RT-DETR a practical baseline for engineering-oriented EL inspection.

Building on this foundation, visual state-space models (e.g., Mamba) offer an alternative paradigm for long-range dependency modeling with linear complexity, which is promising for improving cross-region consistency in EL feature representation [23,24]. However, recursive aggregation in deep state-space modeling may attenuate high-frequency details and cause response diffusion, especially under strong structured background interference where subtle defect cues are easily degraded [25]. Motivated by recent efforts to strengthen local modeling and receptive-field organization (e.g., DSWMamba) [26], this paper proposes MSW-Mamba-Det, an end-to-end framework for PV module EL defect detection. Built upon RT-DETR, MSW-Mamba-Det enhances feature extraction and multi-scale fusion to better match EL imaging characteristics. The design targets two goals: (i) improving cross-region context aggregation and (ii) preserving fine-grained spatial cues for weak and elongated defects, while maintaining a streamlined end-to-end inference process. The decoder continues to adopt a set prediction mechanism to directly output defect categories and bounding boxes.

The main contributions are summarized as follows:

- **MSW-Mamba-Det framework.** We propose an end-to-end detection framework built upon RT-DETR, tailored to PV EL imagery with high resolution, low contrast, and strong structured patterns. The framework improves representation stability while preserving a streamlined inference pipeline.
- **MSW-Mamba module.** We design a multi-scale windowed state-space module with anisotropic scanning strategies to enhance cross-region context aggregation and strengthen modeling of directional defect structures.
- **DetailAware module.** We propose a lightweight detail compensation module that enhances fine-grained textures and edges via local enhancement and adaptive gated fusion, mitigating detail attenuation in deep feature transformations without relying on complex post-processing.
- **PAFB fusion strategy.** We introduce a pyramid attention fusion block that performs attention-driven interaction between adjacent scales and adaptive fusion within a unified semantic space, improving multi-scale consistency and localization stability.
- **Extensive validation.** Comparative experiments, ablation studies, and feature visualization on two public datasets demonstrate the effectiveness of the proposed design in terms of feature focus, background suppression, and cross-scale stability.

The remainder of this paper is organized as follows. Section 2 reviews related work on defect detection for crystalline silicon PV modules. Section 3 presents the proposed method. Section 4 describes the datasets, comparative studies, ablation studies, and visualization analysis. Section 5 concludes the paper and outlines future directions.

## 2. Related Work

### 2.1. Transformer-Based Methods for PV EL Defect Detection

Although Vision Transformers provide strong global context modeling, PV EL images contain pronounced structured patterns—such as metal fingers, busbars, and cell boundaries—that can cause representation entanglement between defects and regular background structures. As a result, local texture and edge cues may be attenuated during deep feature aggregation, which degrades the continuity modeling and precise localization of elongated and low-contrast defects [27]. Within the end-to-end DETR paradigm, several variants have been introduced for PV EL inspection. CSPD-DETR enhances detail preservation through expansion re-parameterization and wavelet-based fusion, partially alleviating detail loss. However, separating regular responses to structured backgrounds from anomalous defect responses remains challenging under strong repetitive textures [28]. PD-DETR improves convergence behavior and alleviates sparse supervision through hybrid matching strategies, yet boundary fidelity for elongated defects can still be sensitive to the intrinsic separability of features when strong structural interference exists [29]. In addition to DETR-style detectors, attention and context modeling mechanisms have been widely explored in single-stage frameworks (e.g., You Only Look Once (YOLO) variants) to enhance weak-defect perception. Wang et al. improved small-object responses with contextual attention, but increased feature interaction can amplify background-induced false positives and place higher requirements on localization stability [30]. Ma et al. introduced pyramid segmentation attention (PSA) to partially preserve spatial resolution, while long-range correlations may still be dominated by repetitive background patterns, leading to representational bias in EL imagery [31]. C2DEM-YOLO incorporates lightweight EMA attention to improve detection sensitivity, yet maintaining continuity and refining boundaries for extremely weak elongated defects remains difficult [32]. For complex backgrounds and irregular defect morphologies, Su et al. proposed BAF-Detector with a bidirectional pyramid to reduce deep-layer feature degradation [33], and Tan et al. employed deformable attention to improve morphological adaptability [34]. Other works integrate additional priors, such as topological/structural knowledge, to support inference for structured defects [35]. LD-DETR simplifies the pipeline by removing the encoder, improving efficiency, but challenges remain in simultaneously maintaining cross-region consistency and preserving local details under texture-dense structured backgrounds [36].

Overall, despite steady progress, existing Transformer- and attention-based approaches still face difficulties in robustly modeling long-range continuity while preventing local detail degradation in PV EL imagery with strong structured interference. This motivates alternative paradigms for long-range modeling with lower computational complexity. In this context, structured state-space models (SSMs), represented by the Mamba architecture, have recently attracted increasing attention due to their linear-complexity sequence modeling capability.

### 2.2. State-Space Models (Mamba) for Vision

To adapt state-space models (SSMs) to 2D vision tasks, existing visual Mamba research has mainly progressed along two directions: (i) designing serialization/scanning strategies for 2D features and (ii) enhancing feature representations to compensate for detail attenuation. However, many studies are still primarily validated on generic vision benchmarks, and their behavior under structured, low-contrast industrial imagery remains less explored [37]. On the serialization and scanning side, VisionMamba adopts bidirectional scanning to strengthen global context aggregation, yet block-wise serialization can partially disrupt spatial adjacency and continuity [38]. VMamba introduces SS2D along with multi-directional cross-scan to reduce information loss induced by naïve row-wise flattening, but robustness under texture-dense structured backgrounds may still be limited due to correlation bias [39]. EfficientVMamba reduces redundant computation via atrous (dilated) scanning; nevertheless, skip sampling may weaken the continuous representation of extremely weak edge signals, which is undesirable for faint linear defects [40]. These efforts highlight the importance of scan-path design, but also indicate that preserving local topology and maintaining continuity remain challenging when the visual signal is weak and the background is highly repetitive. On the feature enhancement side, LocalMamba introduces windowed selective scanning to reinforce local dependencies and improve texture/edge fidelity, while window partitioning can weaken cross-region consistency constraints if long-range interaction is not sufficiently preserved [41]. TinyViM incorporates frequency-domain fusion or low-/high-frequency decomposition to alleviate low-frequency bias and response diffusion in SSM representations, although hybrid branches and operators often increase architectural complexity and training burden [42]. Meanwhile, MambaOut reports that performance gains from SSMs may be unstable on certain vision classification settings, implying that their advantages may be more pronounced in long-sequence dense prediction problems such as detection and segmentation [43]. For detection-oriented applications, Mamba-YOLO embeds SSM blocks into YOLO backbones and introduces local feature enhancement designs (e.g., LSBlock and RGBlock), demonstrating that SSM-based modeling can improve detection accuracy while maintaining efficiency [44]. Beyond 2D imagery, PointMamba targets point clouds, and its space-filling-curve-based traversal provides useful insights for preserving local topological continuity, which is relevant when designing scan paths for 2D structured patterns [45]. DSWMamba, developed for scratch detection on asphalt pavement, proposes a Deep Fusion Selective Scan (DFSS) module to strengthen local modeling and reorganize receptive fields, effectively alleviating localization ambiguity for elongated anisotropic defects [26]. These studies collectively suggest that scan-path organization, local detail preservation, and task-specific fusion strategies are critical for leveraging SSMs in defect detection.

In summary, existing visual Mamba studies provide useful inspiration for PV EL defect inspection, but important limitations remain. Current scanning strategies often struggle to jointly preserve local spatial continuity and model cross-region dependencies under strong structured background interference. In addition, existing fusion mechanisms are mostly designed for generic vision tasks and lack explicit enhancement of weak defect cues and cross-scale alignment in EL imagery. These limitations motivate the proposed MSW-Mamba-Det, which combines anisotropic multi-scale scanning, detail-aware compensation, and adjacent-scale attention fusion within an end-to-end detection framework.

## 3. Method

### 3.1. Overall Architecture

**Figure 1:**
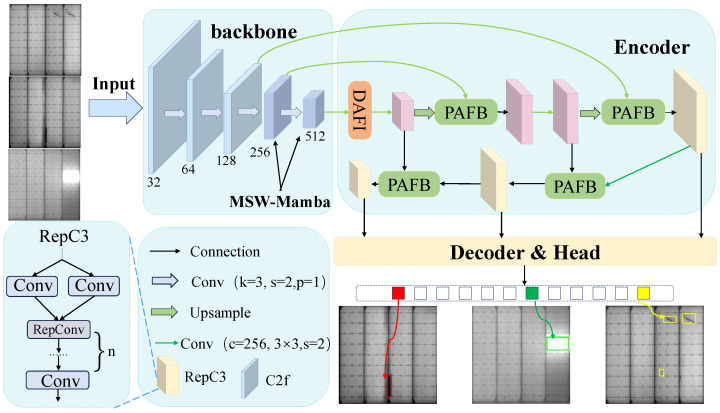
Overall architecture of MSW-Mamba-Det. **DAFI** denotes the neck module, which combines **AIFI** for intra-scale feature interaction and **DetailAware** for detail enhancement.

We propose MSW-Mamba-Det, an end-to-end framework for defect detection in PV module electroluminescence (EL) images, built upon RT-DETR. As illustrated in Figure 1, the framework consists of three main components: (1) an MSW-Mamba backbone for multi-scale feature extraction, (2) an encoder/neck for feature interaction and multi-scale fusion, and (3) a decoder/head for final prediction. Given an input EL image, the MSW-Mamba (Multi-Scale Window Mamba) backbone extracts hierarchical features at multiple scales, which are then fed into the encoder. To mitigate the loss of fine-grained cues that can occur during deep feature transformation, a DetailAware module is integrated in the encoder to enhance high-frequency details and improve the representation of weak and thin defects. Next, high-level features are refined through AIFI (At-Same-Scale Feature Interaction), which performs self-attention within each scale to strengthen semantic discrimination. The resulting multi-scale features are further fused by PAFB (Pyramid Attention Fusion Block), which aggregates adjacent-scale information to reduce semantic gaps and improve localization across scales. Finally, a Transformer decoder performs iterative decoding driven by object queries and outputs a set of predictions, each consisting of a class label and a bounding box.

### 3.2. MSW-Mamba Module

**Figure 2:**
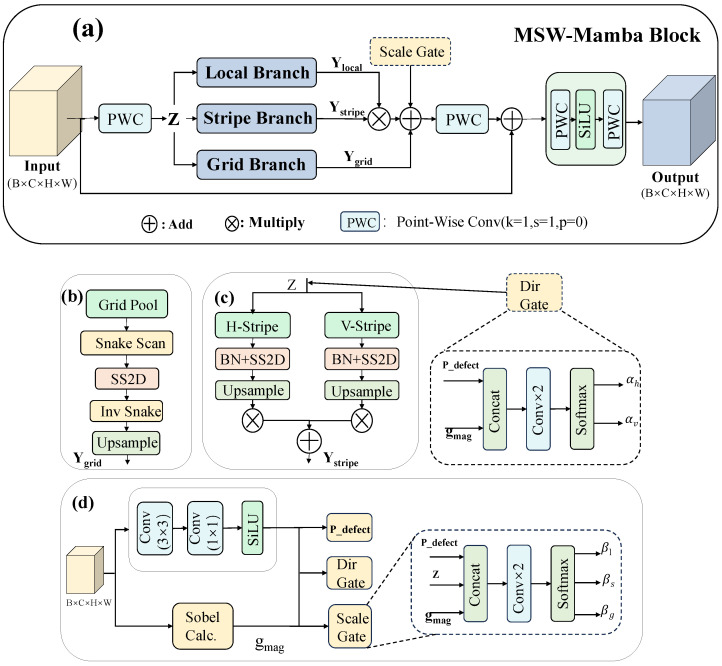
Architecture of the proposed MSW-Mamba module. (**a**) Overall structure of MSW-Mamba. (**b**) Grid branch. (**c**) Stripe branch and directional gate. (**d**) Scale gating driven by the defect saliency map $P_{\mathrm{defect}}$ and gradient-magnitude map $g_{\mathrm{mag}}$.

The standard visual Mamba design (e.g., VMamba) encodes 2D features using a fixed cross-scan strategy, which can be insufficient for EL inspection scenarios where defects exhibit strong anisotropy (e.g., elongated scratches) and require cross-regional continuity reasoning under structured backgrounds. To better capture multi-scale context and directional dependencies, we propose MSW-Mamba (Multi-Scale Window Mamba), as illustrated in Figure 2. MSW-Mamba builds three complementary branches on a shared channel bottleneck and fuses them via an adaptive gated mechanism, enabling unified modeling of local details, anisotropic structures, and coarse global context. In the backbone, MSW-Mamba is inserted at the P4/16 and P5/32 stages, where larger receptive fields are more beneficial for modeling long-range defect continuity and cross-region context.

Given an input feature map $X\in R^{B\times C\times H\times W}$, MSW-Mamba first applies a 1×1 point-wise convolution to obtain an intermediate feature $Z\in R^{B\times C_{\mathrm{mid}}\times H\times W}$. This channel bottleneck preserves spatial resolution and reduces computation before applying the selective state-space operator (SS2D). In our implementation, the SS2D state dimension is set to 16 to balance sequence modeling capacity and efficiency. All three branches then perform long-range dependency modeling based on SS2D, but with different spatial partitioning and traversal strategies.

Local branch: The local branch applies SS2D directly on Z to preserve fine-grained textures and weak-contrast cues, producing a stable local-to-mid-range representation. This branch serves as the reference path because some subtle EL defects still rely on relatively intact local spatial layouts.

Stripe branch: To explicitly strengthen directional continuity, the stripe branch decomposes Z into 1D sequences along two orthogonal directions (horizontal and vertical). Each directional sequence is encoded by SS2D and then reshaped back to the 2D feature grid. The two directional outputs are then fused with a learnable directional gate, allowing the model to emphasize the dominant orientation of anisotropic defects and improve localization consistency for elongated structures.

Grid branch: To capture broader semantics with lower cost, the grid branch first pools Z into a coarse grid representation $Z_{g}\in R^{B\times C_{mid}\times G_{h}\times G_{w}}$. In our implementation, we set $G_{h}=G_{w}=4$, which provides a compact global representation and reduces the serialized sequence length to L=16. This downsampling is implemented via adaptive average pooling to aggregate semantic responses while controlling computational cost. To reduce adjacency disruption caused by naive row-major flattening, we introduce a snake-shaped traversal permutation that alternates scanning direction between neighboring rows: even-indexed rows are flattened left-to-right, while odd-indexed rows are flattened right-to-left. The permuted sequence is then processed by SS2D, inversely permuted back to the 2D grid, and upsampled to the original resolution (H,W) using bilinear interpolation. This design helps preserve neighboring continuity during serialization and is therefore better suited to structured EL backgrounds.

(1) $$\pi :\left\{0,\ldots ,G_{h}-1\right\}\times \left\{0,\ldots ,G_{w}-1\right\}\to \left\{0,\ldots ,G_{h}G_{w}-1\right\}.$$

(2) $$\pi \left(i,j\right)=\left\{\begin{array}{cc} iG_{w}+j, & i\;\mathrm{mod}\;2=0,\\ iG_{w}+\left(G_{w}-1-j\right), & i\;\mathrm{mod}\;2=1. \end{array}\right.$$

(3) $$Y_{\mathrm{grid}}=\mathrm{Up}\left(\pi^{-1}\left(\mathrm{SS}2D(\pi \left(\mathrm{BN}\left(Z_{g}\right)\right))\right),\left(H,W\right)\right).$$

Adaptive gated fusion: Since different defect morphologies rely on different contextual cues, fixed-weight fusion may dilute the branch that is most informative for a specific region. MSW-Mamba therefore introduces a dual-prior gating mechanism driven by (i) a defect saliency map predicted by lightweight convolutions and (ii) a gradient-magnitude map computed using Sobel filters to reflect edge strength. These priors are used to generate spatially varying fusion gates (normalized across branches) that enhance defect-relevant responses while suppressing background-induced activations under strong structural patterns. Here, $\bar{X}$ denotes the channel-averaged feature map and ε is a small constant for numerical stability:

(4) $$g_{\mathrm{mag}}=\sqrt{{\left(S_{x}*\bar{X}\right)}^{2}+{\left(S_{y}*\bar{X}\right)}^{2}+\varepsilon }.$$

Finally, the fused feature is projected back to *C* channels via a 1×1 convolution and stabilized using residual connections with DropPath regularization. A lightweight MLP (implemented by two 1×1 convolutions with a nonlinearity) is further applied for channel-wise mixing, following a Transformer-like block formulation. To reduce parameter redundancy, SS2D parameters are shared across the three branches by default; thus, the performance gains mainly arise from multi-scale partitioning and the proposed adaptive gating rather than simply increasing model capacity.

(5) $$Y=Y+\mathrm{DropPath}\;\left({\mathrm{Conv}}_{1\times 1}^{\left(2\right)}\;\left(\phi \;\left({\mathrm{Conv}}_{1\times 1}^{\left(1\right)}\left(Y\right)\right)\right)\right).$$

### 3.3. DetailAware Module

**Figure 3:**
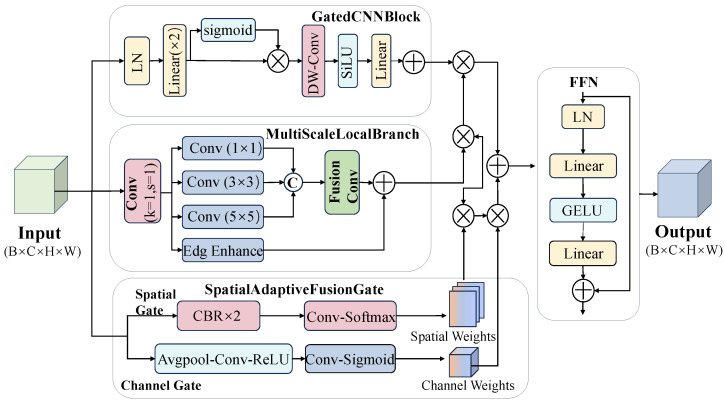
DetailAware Module architecture.

Although state-space models (SSMs) are effective for long-range dependency modeling, deep SSM-based feature transformations may attenuate high-frequency components, thereby weakening thin edges and low-contrast defect cues in EL imagery. To alleviate this issue, we introduce a lightweight DetailAware module before AIFI. Its purpose is not to replace semantic modeling, but to compensate for local detail loss while keeping the overall feature representation stable. As illustrated in Figure 3, the module consists of a local detail enhancement branch, a global semantic preservation branch, pixel-wise adaptive fusion, and post-fusion refinement.

Before entering DetailAware, the input feature is first channel-aligned to C=256. This unified channel width keeps the module compatible with the neck design and controls computational cost.

**Local branch.** The local branch adopts a lightweight multi-scale parallel design to enhance defect-related high-frequency responses. Specifically, the aligned feature is divided into three parallel paths with channel allocation C/4, C/2, and C/4, corresponding to a 1×1 convolution, a 3×3 convolution, and a branch with an effective 5×5 receptive field, respectively. For C=256, the three paths use 64, 128, and 64 channels. This allocation intentionally assigns more channels to the 3×3 path, because medium-scale edge continuity is particularly important for elongated and weak PV defects, while the 1×1 and effective 5×5 paths mainly provide complementary texture and boundary cues. In addition, an edge enhancement path based on depthwise separable 3×3 convolution is introduced to explicitly reinforce edge responses with limited overhead. In this way, the local branch forms a progressive representation from fine textures to edge continuity and slightly broader boundary perception without relying on heavy large-kernel convolutions.

**Global branch.** The global branch is implemented using a lightweight GatedCNNBlock to preserve semantic stability and suppress background interference. It combines layer normalization, linear projection, gating, depthwise separable convolution, and residual connection to filter informative semantic responses while maintaining global structural consistency. This branch is introduced because blindly strengthening local details may also amplify structured background patterns in EL images, such as busbars and cell boundaries.

**Pixel-wise adaptive fusion.** The two branches are fused by a pixel-wise adaptive gating mechanism rather than fixed-weight summation. Specifically, the input feature is passed through a “channel compression →3×3 spatial context modeling →1×1 projection” pipeline to generate a two-channel spatial weight map, followed by softmax normalization along the branch dimension:

(6) $$W_{\mathrm{spatial}}=\mathrm{Softmax}\;\left({\mathrm{Conv}}_{1\times 1}\left({\mathrm{Conv}}_{3\times 3}\left({\mathrm{Conv}}_{1\times 1}\left(X\right)\right)\right)\right)\in R^{B\times 2\times H\times W}.$$

The fused feature is then computed as

(7) $$F_{\mathrm{fused}}=W_{\mathrm{spatial}}^{\left(0\right)}⊙F_{\mathrm{global}}+W_{\mathrm{spatial}}^{\left(1\right)}⊙F_{\mathrm{local}}.$$

This design allows the module to adaptively determine whether each spatial location should rely more on detail enhancement or semantic preservation, which is more suitable for EL defects with highly uneven local visibility.

**Post-fusion refinement.** After fusion, the feature is refined by channel attention and a feed-forward network (FFN). Channel attention recalibrates defect-relevant channels through global average pooling and lightweight convolution, while the FFN uses a hidden dimension of 1024 to enhance nonlinear representation capacity. Residual connections are retained throughout to stabilize optimization:

(8) $$F_{\mathrm{out}}=\left(F_{\mathrm{fused}}⊙\sigma \left(\mathrm{Conv}(\mathrm{GAP}\left(F_{\mathrm{fused}}\right))\right)\right)+\mathrm{FFN}\left(F_{\mathrm{fused}}\right).$$

Overall, DetailAware is designed as a lightweight compensation module for detail attenuation in deep SSM-based representations. By combining explicit local enhancement, semantic preservation, and pixel-wise adaptive fusion under a unified 256-channel setting, it provides AIFI with sharper defect boundaries and more reliable local cues while keeping the added computational burden moderate.

### 3.4. PAFB Module

**Figure 4:**
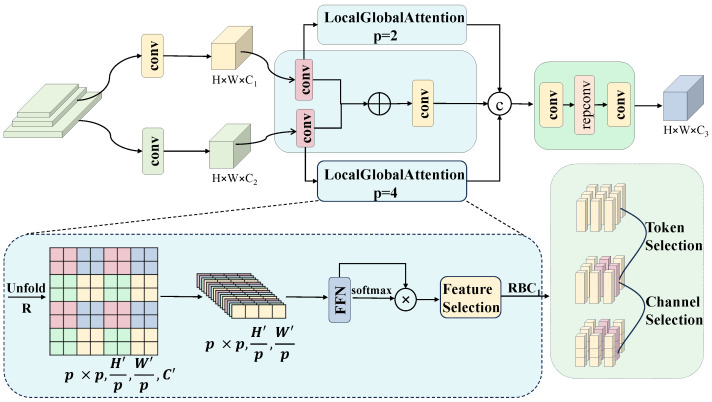
PAFB Module architecture.

Features extracted from different backbone stages are complementary: shallow features preserve rich spatial details but have limited semantic abstraction, whereas deep features provide stronger semantic discrimination but at a reduced spatial resolution. To better exploit cross-scale complementarities, inspired by the hierarchical fusion strategy in HCF-Net [46], we introduce PAFB, which performs hierarchical attention-based interaction between adjacent-scale features, as illustrated in Figure 4.

PAFB takes two feature maps from adjacent stages, denoted by $F_{\mathrm{low}}$ and $F_{\mathrm{high}}$. First, both features are channel-aligned to C=256 through 1×1 convolutions, followed by a 3×3 convolution to produce a baseline fusion feature $F_{\mathrm{base}}$. This baseline acts as a stable anchor for subsequent hierarchical enhancement, helping preserve the original cross-scale information during fusion. For notational convenience, the channel-aligned feature stream corresponding to each adjacent-scale input is denoted by $X_{i}$, where i∈{low,high}.

(9) $$F_{i}^{\mathrm{attn}}=\mathrm{Softmax}\;\left(\mathrm{MLP}({\mathrm{Unfold}}_{p}\left(X_{i}\right))\right)⊙\mathrm{MLP}\;\left({\mathrm{Unfold}}_{p}\left(X_{i}\right)\right).$$

Next, each feature stream is processed by a Local–Global Attention (LGA) branch with a dual-scale patch partitioning strategy. In our implementation, the patch sizes are set to p=2 and p=4. The finer partition (p=2) emphasizes local textures and defect edges, whereas the coarser partition (p=4) captures broader structural semantics. This fixed dual-scale setting is adopted as a practical trade-off between fine-detail sensitivity and contextual modeling range while keeping the attention computation lightweight. Concretely, features are unfolded into non-overlapping patches at each scale, projected by an MLP, and then reweighted by self-attention to model dependencies within each patch group. The MLP uses a hidden expansion ratio of 2 to balance representation capacity and parameter efficiency.

To further guide attention toward defect-relevant regions, we introduce a learnable prompt vector that generates a similarity mask. The mask injects top-down semantic guidance into local representations, so that attention responses are enhanced around potential defect regions while background-dominated responses are suppressed. Each feature stream is processed by LGA at both patch scales, and the resulting outputs are concatenated.

(10) $$M=\mathrm{clamp}\;\left(\frac{F_{i}^{\mathrm{attn}}\cdot P}{∥F_{i}^{\mathrm{attn}}∥\;∥P∥},0,1\right).$$

(11) $$F_{i}^{\mathrm{out}}=\left(F_{i}^{\mathrm{attn}}⊙M\right)W_{td}^{l}.$$

Here, *P* is the learnable prompt and *M* is the similarity mask. Finally, the hierarchical attention features are concatenated with the baseline feature, followed by channel compression and re-parameterizable convolution (RepConv) for feature reorganization. During training, RepConv adopts a multi-branch structure including a 3×3 convolution, a 1×1 convolution, and an identity branch. These branches are structurally re-parameterized into a single 3×3 convolution at inference time, thereby improving execution efficiency without changing the inference structure.

Overall, PAFB enhances cross-scale feature fusion by combining hierarchical attention (local texture + global semantics) with efficient feature reorganization, providing the detection head with multi-scale representations that are both spatially detailed and semantically discriminative.

## 4. Experiments

### 4.1. Datasets

This study employs two publicly available datasets for PV module defect detection, namely PV-Multi-Defect-main [47] and PVEL-AD [48], for model training, validation, and testing. The two datasets are complementary in defect taxonomy and image characteristics (e.g., resolution and defect distribution), enabling evaluation under multiple inspection scenarios.

**Figure 5:**
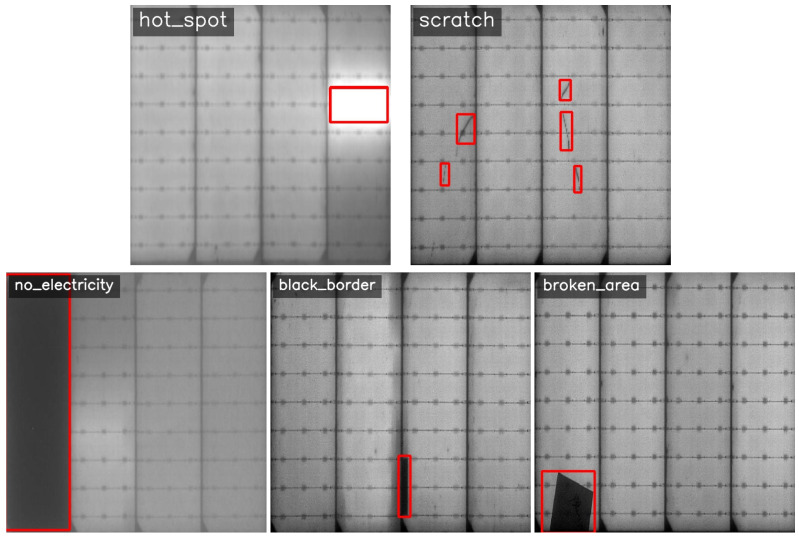
Representative defect examples from PV-Multi-Defect-main.

As shown in Figure 5, PV-Multi-Defect-main contains 1105 EL images of PV modules. All images are resized to 600×600 pixels. The dataset covers five defect categories with detailed bounding-box annotations: 2079 hot_spot instances, 1367 scratches, 181 no_electricity cases, 256 black_border instances, and 98 broken_area samples. From a morphological perspective, scratches and hot_spots typically appear as small-scale or elongated patterns, requiring strong fine-grained feature representation and precise localization. In contrast, no_electricity and black_border defects often occupy larger regions with more salient texture or intensity variations, making them comparatively easier to identify based on contour and grayscale differences.

**Figure 6:**
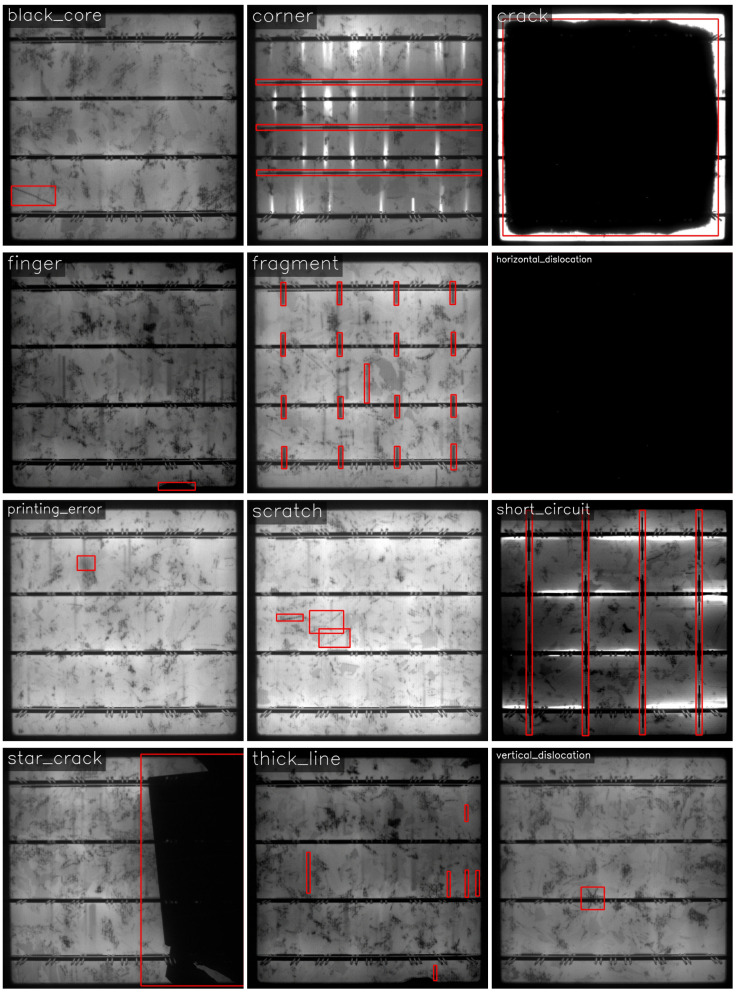
Representative defect examples from PVEL-AD.

As shown in Figure 6, PVEL-AD was jointly released by Hebei University of Technology and Beihang University. As a large-scale benchmark dataset for PV module defect detection, it provides substantial diversity in both defect types and visual appearances. It contains 4601 EL images, each with a resolution of 1024×1024 pixels, and includes 12 defect categories: 1035 black_cores, 36 corners, 1301 cracks, 2999 fingers, 30 fragments, 798 horizontal_dislocations, 478 printing_errors, 54 scratches, 492 short_circuits, 139 star_cracks, 981 thick_lines, and 137 vertical_dislocations. Notably, the dataset includes a large number of fine-grained defects with high intra-class variability, providing a rigorous test bed for assessing model robustness under complex inspection conditions.

In this study, each dataset is partitioned into training, validation, and test subsets at a ratio of 7:1.5:1.5 using a stratified split at the image level based on the dominant class of each annotated image. Specifically, PV-Multi-Defect-main contains 773, 166, and 166 images in the training, validation, and test subsets, respectively, while PVEL-AD contains 3220, 690, and 691 images in the corresponding subsets. The partitioning yields broadly consistent class proportions across the three subsets, and detailed per-split class-instance statistics for both datasets are provided in the Supplementary Materials. To avoid direct data leakage, each image and its corresponding annotation file is assigned exclusively to one subset during dataset organization. The validation set is used for hyperparameter tuning and early stopping based on validation performance to mitigate overfitting.

### 4.2. Experimental Setup and Evaluation Metrics

The experiments were conducted on a workstation equipped with an AMD Ryzen Threadripper 3970X CPU (Advanced Micro Devices, Inc., Santa Clara, CA, USA), two NVIDIA GeForce RTX 3080 GPUs (NVIDIA Corporation, Santa Clara, CA, USA) (12 GB VRAM each), and Ubuntu 22.04. The software environment included Python 3.9, PyTorch 2.0.1, CUDA 11.8, cuDNN 8.5, Ultralytics 8.20.1, MMDetection (MMDet) 3.3, and MMCV 2.1. Unless otherwise stated, all methods were trained and evaluated under the same protocol for fair comparison. The default training schedule used an input resolution of 640×640, the SGD optimizer, an initial learning rate of 0.01, a maximum of 500 training epochs, a batch size of 4, and four data-loading workers. Early stopping was applied based on validation performance, and the best-performing checkpoint on the validation set was used for testing. Unless explicitly specified, the same training schedule, early-stopping strategy, and evaluation setting were used for both the proposed method and the compared detectors.

For reproducibility, the module-specific settings of the proposed method were kept fixed across all experiments. Specifically, MSW-Mamba was inserted at the P4/16 and P5/32 stages, with the SS2D state dimension set to 16 and the grid branch using $G_{h}=G_{w}=4$; DetailAware used channel alignment to 256 dimensions, local-branch channel allocation of C/4, C/2, and C/4, and an FFN hidden dimension of 1024; and PAFB aligned adjacent-scale features to 256 channels and adopted dual-scale Local–Global Attention with patch sizes p=2 and p=4 and an MLP expansion ratio of 2.

To comprehensively evaluate detection performance, we adopt the standard COCO metrics [49]. We report overall average precision (AP) as well as class-wise AP. Specifically, $AP_{50:95}$ denotes the mean AP averaged over IoU thresholds from 0.50 to 0.95 with a step size of 0.05, while $AP_{50}$ denotes the AP at an IoU threshold of 0.50. In addition, $AP_{s}$, $AP_{m}$, and $AP_{l}$ evaluate detection performance on small, medium, and large objects, respectively. Precision (P) and recall (R) are also reported. Model complexity and runtime efficiency are measured in terms of the number of parameters (Params), GFLOPs, and frames per second (FPS). For fairness, all FPS results were measured under the same hardware and software environment using the same input resolution and inference setting for all compared models.

### 4.3. Comparative Study

To evaluate the effectiveness of the proposed method, we compared it with 12 representative baselines under identical experimental conditions on the PV-Multi-Defect-main and PVEL-AD datasets. The compared baselines include Faster R-CNN [50], an EfficientNet-based baseline [51], DCNv2 [52], DyHead [53], TOOD [54], YOLOv8m [55], YOLOv9t [56], YOLOv10b [57], YOLO11m [58], YOLO12m [59], Mamba-YOLO [44], and RT-DETR [22]. All models were trained and evaluated using identical data splits, training schedules, early-stopping strategies, and evaluation settings unless otherwise specified.

RT-DETR serves as a practical end-to-end baseline with competitive accuracy and efficiency among the compared detectors. Building on this baseline, MSW-Mamba-Det further improves detection performance on both datasets.

Table 1 summarizes the results on PV-Multi-Defect-main. MSW-Mamba-Det achieves the highest $AP_{50:95}$ (60.4%) and $AP_{50}$ (89.4%) among all evaluated methods, demonstrating improved detection quality under both strict and lenient localization tolerances. Compared with RT-DETR, MSW-Mamba-Det improves ${\mathrm{AP}}_{50:95}$ by 2.5 points (from 57.9% to 60.4%) and $AP_{50}$ by 5.7 points (from 83.7% to 89.4%), indicating that the proposed architecture enhances feature representation for EL images under strong structured background interference.

In terms of scale-specific performance, MSW-Mamba-Det attains ${\mathrm{AP}}_{m}$ of 56.0% and ${\mathrm{AP}}_{l}$ of 69.1%, improving over RT-DETR by 1.6 points (from 54.4% to 56.0%) and 2.4 points (from 66.7% to 69.1%), respectively. For small objects, MSW-Mamba-Det achieves an ${\mathrm{AP}}_{s}$ of 34.7%, improving over RT-DETR by 1.8 points (from 32.9% to 34.7%). Although YOLOv8m achieves a slightly higher ${\mathrm{AP}}_{s}$ (36.4%), it comes with substantially higher computational cost and parameter count.

Overall, these results indicate that MSW-Mamba-Det improves defect detection across object scales. Compared with RT-DETR, this accuracy gain is accompanied by increased computational cost and a moderate reduction in inference speed. Specifically, on PV-Multi-Defect-main, GFLOPs and parameters increase from 57.0 to 68.0 and from 19.9 M to 26.1 M, respectively, while FPS decreases from 114.7 to 99.4 under the same testing setting. Combined with the ablation results, this suggests that the added overhead mainly comes from the introduced context modeling and adjacent-scale fusion operations. At the same time, the proposed method is not uniformly superior in every subcase: for example, YOLOv8m still achieves a slightly higher $AP_{s}$. Therefore, the advantage of MSW-Mamba-Det is more evident in scenarios where structured-background suppression, cross-region context modeling, and boundary consistency are more critical, and the proposed method is more suitable for accuracy-oriented PV EL inspection scenarios where robustness and localization stability are prioritized over minimal model cost. Nevertheless, compared with higher-cost baselines such as DCNv2 and TOOD, our method achieves higher AP at lower complexity, while still maintaining efficient real-time inference, indicating more effective modeling of structured backgrounds and multi-scale defect patterns in EL imagery.

**Table 1:** Comparison with representative detectors on PV-Multi-Defect-main.

Model	PV-Multi-Defect-Main (Detection Metrics)							Complexity/Efficiency		
	${\mathrm{AP}}_{50:95}$	${\mathrm{AP}}_{50}$	**P**	**R**	${\mathrm{AP}}_{s}$	${\mathrm{AP}}_{m}$	${\mathrm{AP}}_{l}$	**GFLOPs**	**Params (M)**	**FPS**
Faster-RCNN [50]	49.9	82.5	**88.3**	77.0	18.6	45.9	44.4	69.9	28.3	–
EfficientNet [51]	32.0	63.1	59.5	68.0	12.5	36.7	28.4	54.9	19.6	–
DCNv2 [52]	58.8	88.5	86.9	**86.5**	30.9	53.1	60.6	80.2	42.2	–
DyHead [53]	52.4	85.5	82.7	78.3	20.3	53.9	52.7	43.6	38.9	–
TOOD [54]	45.3	77.3	77.3	75.8	23.4	36.0	43.0	80.4	32.2	–
YOLOv8m [55]	56.1	82.8	80.7	77.2	**36.4**	49.7	55.0	78.7	25.8	**130.7**
YOLOv9t [56]	57.2	84.9	81.4	80.0	23.4	53.1	62.6	77.9	20.2	101.8
YOLOv10b [57]	56.8	83.2	84.8	75.6	33.9	49.1	55.7	91.6	19.0	106.2
YOLO11m [58]	56.3	83.2	84.7	74.3	19.2	43.2	48.5	67.7	20.0	115.1
YOLO12m [59]	53.3	82.8	81.1	77.6	15.4	46.7	55.1	59.5	19.6	85.2
Mamba-YOLO [44]	46.1	75.2	75.2	65.6	15.2	39.1	50.9	49.7	21.8	94.9
RT-DETR [22]	57.9	83.7	81.4	80.7	32.9	54.4	66.7	57.0	19.9	114.7
Ours	**60.4**	**89.4**	85.5	86.3	34.7	**56.0**	**69.1**	68.0	26.1	99.4

Note: Values in boldface represent the best performance for each metric, and underlined values represent the second-best performance.

Table 2 reports results on PVEL-AD, which contains higher-resolution images (1024×1024 pixels) and more diverse fine-grained defect types. Compared with PV-Multi-Defect-main, most detectors exhibit larger performance variations on this dataset. MSW-Mamba-Det still achieves the best overall metrics, suggesting stronger robustness under more complex defect appearances and richer category distributions. Specifically, MSW-Mamba-Det reaches $AP_{50:95}$ of 68.0% and $AP_{50}$ of 94.2%, indicating a good balance between localization accuracy and detection sensitivity. On small defects, MSW-Mamba-Det achieves ${\mathrm{AP}}_{s}$ of 36.1%, second only to YOLO12m (37.2%), and notably higher than RT-DETR (30.1%) and Mamba-YOLO (32.6%). For medium and large defects, MSW-Mamba-Det achieves ${\mathrm{AP}}_{m}$ of 60.4% and ${\mathrm{AP}}_{l}$ of 66.7%, reflecting improved context aggregation and boundary consistency modeling. Overall, the proposed method maintains competitive performance across ${\mathrm{AP}}_{s}$, ${\mathrm{AP}}_{m}$, and ${\mathrm{AP}}_{l}$, but its advantage is more evident on medium and large defects and under more complex structural backgrounds. Although YOLO12m achieves a slightly higher ${\mathrm{AP}}_{s}$, MSW-Mamba-Det shows stronger overall robustness when defect localization requires cross-scale semantic alignment and more stable boundary representation in high-resolution EL imagery.

Taken together, these results suggest that the proposed method provides more consistent advantages in PV EL inspection scenarios characterized by strong structured backgrounds, weak-contrast responses, and defects requiring both long-range continuity modeling and cross-scale alignment. Its gains are especially stable on medium and large defects, whereas in some small-defect settings, lighter YOLO-style detectors may still show slightly stronger AP_s_. Therefore, MSW-Mamba-Det is better suited to accuracy-oriented EL inspection scenarios where robustness and localization stability are prioritized.

Table 3 compares backbone networks under the same detection head, training strategy, and input resolution, demonstrating the effectiveness of the proposed MSW-Mamba backbone. Specifically, MSW-Mamba is evaluated against EfficientViT [60], ConvNeXtV2 [61], Swin Transformer [27], VisionMamba [38], and MambaOut [43]. Among CNN-based backbones, ConvNeXtV2 attains relatively high precision, yet yields the lowest overall $AP_{50:95}$ (53.8%). This suggests that representations dominated by local convolutional patterns are less effective at capturing the long-range continuity and global context required to distinguish complex defects from structured background patterns in EL imagery. For the Transformer-based backbone (Swin Transformer), although the computational cost increases substantially (97.0 GFLOPs and 36.3M parameters), $AP_{50:95}$ only reaches 55.2%, indicating a limited accuracy gain relative to the increased cost. This implies that simply increasing attention computation does not necessarily translate into more discriminative features in low-contrast EL images with strong structured priors. Visual state-space model (SSM) backbones provide a stronger overall trade-off. VisionMamba and MambaOut achieve ${\mathrm{AP}}_{50:95}$ of 57.9% and 57.0%, respectively, outperforming most CNN and Transformer counterparts. This highlights the advantage of SSM-based backbones in modeling long-range dependencies with linear-time complexity. Notably, the proposed MSW-Mamba achieves the best performance, reaching 58.6% ${\mathrm{AP}}_{50:95}$ and 87.8% $AP_{50}$. It also maintains a balanced precision–recall trade-off (P = 87.1%, R = 81.6%), indicating effective suppression of background-induced false responses while consistently improving detection performance in EL inspection scenarios characterized by weak contrast and strong structural patterns.

To further verify that the reported gains are not caused by random initialization, we repeated the baseline RT-DETR and the proposed MSW-Mamba-Det with five different training random seeds under otherwise identical settings while keeping the dataset split fixed. As shown in Table 4, the proposed method consistently outperforms RT-DETR on both datasets with stable improvements in $AP_{50}$ and $AP_{50:95}$. On PV-Multi-Defect-main, MSW-Mamba-Det improves $AP_{50:95}$ from 57.8±0.3% to 60.2±0.3%, while on PVEL-AD it improves $AP_{50:95}$ from 65.7±0.5% to 67.8±0.2%. Welch’s *t*-test on $AP_{50:95}$ further supports the statistical significance of the observed gains (p<0.001 on both datasets).

**Table 2:** Comparison with representative detectors on PVEL-AD.

Model	PVEL-AD (Detection Metrics)							Complexity/Efficiency		
	${\mathrm{AP}}_{50:95}$	${\mathrm{AP}}_{50}$	**P**	**R**	${\mathrm{AP}}_{s}$	${\mathrm{AP}}_{m}$	${\mathrm{AP}}_{l}$	**GFLOPs**	**Params (M)**	**FPS**
Faster-RCNN [50]	41.9	64.3	75.1	54.1	27.8	44.7	34.4	69.9	28.3	–
EfficientNet [51]	51.2	77.6	80.3	73.2	23.5	57.3	53.2	54.9	19.6	–
DCNv2 [52]	55.4	78.3	73.5	76.8	25.5	55.8	49.4	80.2	42.2	–
DyHead [53]	58.7	93.0	85.0	88.8	31.9	58.8	55.9	43.6	38.9	–
TOOD [54]	42.4	76.2	78.9	67.8	22.1	42.7	36.0	80.4	32.2	–
YOLOv8m [55]	62.9	91.8	87.5	90.3	26.5	58.6	59.8	78.7	25.8	**130.7**
YOLOv9t [56]	64.2	91.9	88.0	89.2	24.9	**61.2**	61.7	77.9	20.2	101.8
YOLOv10b [57]	59.6	81.9	78.1	77.6	29.6	56.6	56.2	91.6	19.0	106.2
YOLO11m [58]	61.5	91.8	86.6	91.4	29.7	54.3	61.9	67.7	20.0	115.1
YOLO12m [59]	59.7	89.6	79.2	**91.9**	**37.2**	57.4	57.0	59.5	19.6	85.2
Mamba-YOLO [44]	66.8	93.3	88.3	90.6	32.6	50.7	63.6	49.7	21.8	94.9
RT-DETR [22]	65.8	92.4	91.1	84.7	30.1	45.2	61.6	57.0	19.9	114.7
Ours	**68.0**	**94.2**	**93.9**	90.5	36.1	60.4	**66.7**	68.0	26.1	99.4

Note: Values in boldface represent the best performance for each metric, and underlined values represent the second-best performance.

### 4.4. Ablation Studies

To quantify the individual contribution of each proposed module, we conducted ablation studies on both PV-Multi-Defect-main and PVEL-AD under strictly identical settings, including the same RT-DETR baseline, data split, input resolution, optimizer, training schedule, early-stopping strategy, and evaluation protocol. The only difference across variants is the activation of MSW-Mamba, DetailAware, and PAFB.

**Table 3:** Comparison of different backbones under the same detection head, training strategy, and input resolution.

Backbone	P	R	${\mathrm{AP}}_{50}$	${\mathrm{AP}}_{50:95}$	GFLOPs	Params (M)
EfficientViT [60]	82.4	72.6	82.9	56.8	53.3	17.4
ConvNeXtV2 [61]	84.8	78.3	85.1	53.8	45.3	15.7
Swin Transformer [27]	81.0	77.6	84.0	55.2	97.0	36.3
VisionMamba [38]	81.2	83.2	84.1	57.9	53.7	16.1
MambaOut [43]	81.3	78.2	84.6	57.0	46.6	13.8
MSW-Mamba	87.1	81.6	87.8	58.6	57.1	23.0

**Table 4:** Stability analysis over five runs with different training random seeds under identical settings. Results are reported as mean ± standard deviation. The *p*-value is obtained from Welch’s *t*-test on $AP_{50:95}$ between RT-DETR and the proposed method.

Dataset	Model	Runs	P	R	${\mathrm{AP}}_{50}$	${\mathrm{AP}}_{50:95}$	*p*-Value
PV-Multi-Defect-main	RT-DETR	5	81.6 ± 1.1	80.6 ± 1.1	83.4 ± 0.6	57.8 ± 0.3	–
	Ours	5	86.0 ± 1.0	85.6 ± 1.3	89.1 ± 0.5	60.2 ± 0.3	<0.001
PVEL-AD	RT-DETR	5	92.0 ± 0.7	85.5 ± 0.8	92.5 ± 0.5	65.7 ± 0.5	–
	Ours	5	93.7 ± 1.0	90.4 ± 0.4	94.1 ± 0.4	67.8 ± 0.2	<0.001

Table 5 reports the ablation results on PV-Multi-Defect-main under identical training and evaluation settings, where the baseline achieves an $AP_{50:95}$ of 57.9%. When enabled individually, MSW-Mamba improves $AP_{50:95}$ by 0.7 points (from 57.9% to 58.6%) with only a negligible increase in GFLOPs, while $AP_{50}$ increases by 4.1 points (from 83.7% to 87.8%). Precision also rises by 5.7 points (from 81.4% to 87.1%), suggesting that multi-scale windowing and anisotropic modeling mainly enhance feature discriminability and suppress background-induced false positives. When enabled individually, DetailAware improves $AP_{50:95}$ by 1.1 points (from 57.9% to 59.0%) and recall by 4.1 points (from 80.7% to 84.8%), while slightly reducing computation, indicating that detail compensation helps mitigate missed detections by strengthening fine-grained cues. When enabled individually, PAFB improves $AP_{50:95}$ by 0.5 points (from 57.9% to 58.4%) and $AP_{50}$ by 1.9 points (from 83.7% to 85.6%), showing that adjacent-scale fusion alone can already provide additional performance gains. When MSW-Mamba and DetailAware are combined, $AP_{50}$ reaches 88.2% and recall increases to 84.9%, demonstrating their complementary effects on reducing false positives and false negatives. Enabling all three modules yields the best detection accuracy, with $AP_{50:95}$ reaching 60.4% and $AP_{50}$ reaching 89.4%, corresponding to improvements of 2.5 and 5.7 points over the baseline, respectively, together with a more balanced precision–recall trade-off (P = 85.5%, R = 86.3%). These results indicate that PAFB further elevates performance when built upon the stronger representations produced by MSW-Mamba and DetailAware.

**Table 5:** Ablation study on PV-Multi-Defect-main under identical training and evaluation settings.

Baseline	MSW-Mamba	DetailAware	PAFB	P	R	${\mathrm{AP}}_{50}$	${\mathrm{AP}}_{50:95}$	GFLOPs	Params (M)
✓				81.4	80.7	83.7	57.9	57.0	19.9
✓	✓			87.1	81.6	87.8 (+4.1)	58.6 (+0.7)	57.1	23.0
✓		✓		81.5	84.8	86.2 (+2.5)	59.0 (+1.1)	56.6	19.8
✓			✓	82.2	80.3	85.6 (+1.9)	58.4 (+0.5)	68.0	23.2
✓	✓	✓		84.8	84.9	88.2 (+4.5)	59.0 (+1.1)	56.8	23.1
✓	✓	✓	✓	**85.5**	**86.3**	**89.4 (+5.7)**	**60.4 (+2.5)**	68.0	26.1

Note: Boldface values represent the best performance. The checkmark (✓) indicates the adopted module/baseline.

Table 6 reports the ablation results on PVEL-AD under identical training and evaluation settings. As a higher-resolution dataset with more diverse defect types and more intricate fine-grained structures, PVEL-AD provides a more challenging test bed for validating the robustness of each module. The baseline achieves an ${\mathrm{AP}}_{50:95}$ of 65.8%. When enabled individually, MSW-Mamba improves $AP_{50:95}$ by 1.0 points (from 65.8% to 66.8%) and recall by 5.2 points (from 84.7% to 89.9%), indicating that the proposed multi-scale modeling can effectively reduce missed detections under more challenging inspection conditions. When enabled individually, DetailAware improves $AP_{50:95}$ by 1.1 points (from 65.8% to 66.9%) and recall by 6.2 points (from 84.7% to 90.9%), suggesting that detail compensation is particularly beneficial for preserving weak defect cues in high-resolution EL imagery. When enabled individually, PAFB provides a modest gain of 0.2 points in $AP_{50:95}$ (from 65.8% to 66.0%) and 1.2 points in ${\mathrm{AP}}_{50}$ (from 92.4% to 93.6%), showing that adjacent-scale fusion alone can already bring additional improvements. When MSW-Mamba and DetailAware are combined, ${\mathrm{AP}}_{50:95}$ further increases to 67.2%, confirming their complementary effects. Enabling all three modules yields the best detection accuracy, with ${\mathrm{AP}}_{50:95}$ reaching 68.0% and ${\mathrm{AP}}_{50}$ reaching 94.2%, corresponding to improvements of 2.2 and 1.8 points over the baseline, respectively, while precision and recall increase to 93.9% and 90.5%. Overall, these results indicate that PAFB contributes most effectively when operating on the enhanced features produced by MSW-Mamba and DetailAware, helping align cross-scale semantics and stabilize detection under complex structural backgrounds.

In addition to the accuracy gains, we further analyzed the impact of each module on real-time performance under the same 640×640 inference setting. The baseline RT-DETR runs at 114.7 FPS. After introducing MSW-Mamba and DetailAware individually, the inference speed decreases to 110.3 FPS and 109.4 FPS, respectively, indicating that both modules introduce only moderate runtime overhead. The variant with PAFB achieves 102.8 FPS, while the full MSW-Mamba-Det achieves 99.4 FPS. These results show that the proposed modules improve detection accuracy at the cost of a moderate reduction in inference speed, while the final model still maintains efficient real-time inference capability.

**Table 6:** Ablation study on PVEL-AD under identical training and evaluation settings.

Baseline	MSW-Mamba	DetailAware	PAFB	P	R	${\mathrm{AP}}_{50}$	${\mathrm{AP}}_{50:95}$	GFLOPs	Params (M)
✓				91.1	84.7	92.4	65.8	57.0	19.9
✓	✓			92.7	89.9	93.4 (+1.0)	66.8 (+1.0)	57.1	23.0
✓		✓		91.8	90.9	94.7 (+2.3)	66.9 (+1.1)	56.6	19.8
✓			✓	90.4	87.0	93.6 (+1.2)	66.0 (+0.2)	68.0	23.2
✓	✓	✓		92.0	88.5	94.3 (+1.9)	67.2 (+1.4)	56.8	23.1
✓	✓	✓	✓	**93.9**	**90.5**	**94.2 (+1.8)**	**68.0 (+2.2)**	68.0	26.1

Note: Boldface values represent the best performance. The checkmark (✓) indicates the adopted module/baseline.

**Figure 7:**
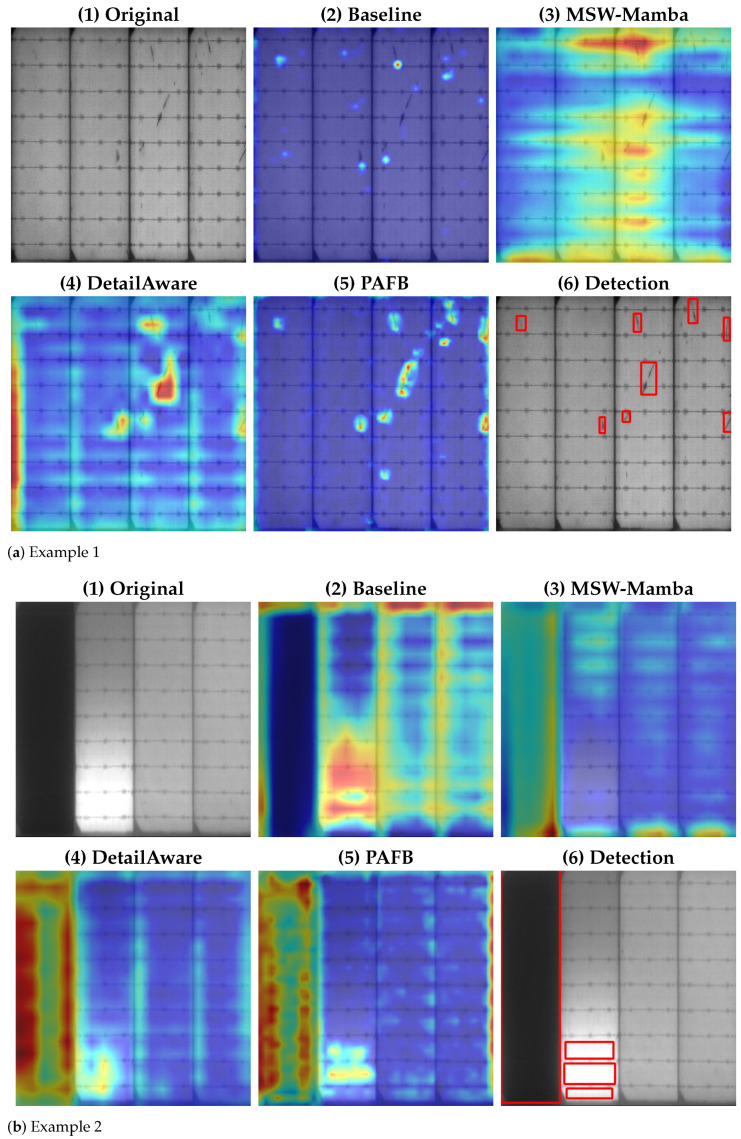
Grad-CAM visualizations and detection results at different stages. For each example, the panels are arranged in the following order: (1) original image, (2) baseline response, (3) MSW-Mamba response, (4) DetailAware response, (5) PAFB response, and (6) final detection result.

Figure 7 provides a qualitative analysis using Grad-CAM visualizations at different network stages, along with the final detections. In the baseline, shallow features mainly show scattered high-response regions, often corresponding to bright spots, noise, and local contrast variations, and they insufficiently capture the continuity of weak-contrast elongated defects. After incorporating MSW-Mamba, the response maps exhibit more coherent stripe-like activations that align with the dominant structural directions of PV cells, indicating strengthened anisotropic modeling and improved long-range context aggregation. With DetailAware, high-response regions become more concentrated around defect areas, while responses to regular background textures are suppressed, consistent with its role in compensating detail loss before decoding. After PAFB fusion, activations become more spatially consistent with defect morphology, and spurious responses confined to a single scale are reduced, suggesting improved cross-scale alignment. Additional examples indicate that under strong structural interference (e.g., pronounced boundary intensity gradients), intermediate responses can be biased toward structural edges; however, after the sequential application of the proposed modules, defect-related activations remain more distinguishable, supporting the robustness of the proposed design in complex EL inspection scenarios.

## 5. Conclusions

EL-based defect inspection for PV modules is important for improving system reliability and safety and reducing inspection and maintenance costs, thereby supporting the large-scale deployment of PV and BIPV systems. Targeting the characteristics of PV EL imagery—high resolution, low contrast, and strong structured patterns—this study proposes MSW-Mamba-Det, a high-accuracy end-to-end framework for crystalline-silicon PV defect detection. The proposed approach addresses key challenges such as precise localization of elongated defects, detecting faint, low-contrast scratches, and robustness under structured background interference.

Built on the RT-DETR end-to-end detection paradigm, MSW-Mamba-Det introduces three complementary modules. First, MSW-Mamba serves as the core representation unit and adopts a Local/Stripe/Grid design to jointly model multi-scale context and anisotropic dependencies, improving long-range continuity modeling for linear defects. Second, the DetailAware module enhances fine-grained cues by selectively compensating high-frequency textures and edges prior to the encoder, alleviating detail attenuation in deep feature transformations. Third, the PAFB module performs cross-scale attention-based fusion to adaptively combine shallow spatial details with deep semantic features within a unified channel space, thereby improving multi-scale fusion effectiveness and detection stability.

Experiments on two public EL datasets demonstrate that MSW-Mamba-Det consistently outperforms representative CNN- and Transformer-based detectors under the same protocol. Compared with RT-DETR, MSW-Mamba-Det improves ${\mathrm{AP}}_{50:95}$ by 2.5 points (from 57.9% to 60.4%) on PV-Multi-Defect-main and by 2.2 points (from 65.8% to 68.0%) on PVEL-AD. The advantages of the proposed method are more consistent in EL inspection scenarios characterized by strong structured backgrounds, weak-contrast defect responses, and defects requiring both long-range continuity modeling and cross-scale alignment. In particular, the gains are more stable on medium and large defects, where contextual aggregation and boundary consistency are more critical. At the same time, the method is not uniformly superior in every subcase, since lighter YOLO-style detectors may still show slightly stronger performance on some small-defect settings. Importantly, the method preserves an end-to-end inference pipeline without additional post-processing. Meanwhile, the improved detection accuracy is accompanied by a moderate increase in computational complexity relative to RT-DETR, indicating that the proposed design is more suitable for accuracy-oriented PV EL inspection scenarios.

Despite the encouraging results, several limitations remain. Future work will focus on reducing parameters and computational cost via pruning and quantization to enable deployment on resource-constrained edge devices. To reduce reliance on exhaustive manual annotations, we will also explore semi-supervised learning with unlabeled data and synthetic defect generation. Finally, we plan to investigate multimodal fusion (e.g., visible and infrared/thermal imaging) to improve robustness under varying operational and environmental conditions.

## Data Availability

The datasets used in this study are publicly available. PV-Multi-Defect-main and PVEL-AD can be obtained from their original publications and/or project pages. Detailed per-split class-instance statistics are provided in the Supplementary Materials, while the processed data splits, training configuration files, and evaluation scripts used to support the findings of this study are available from the corresponding author upon reasonable request.

## Supplementary Materials

The following supporting information can be downloaded at https://www.mdpi.com/article/10.3390/s26092616/s1, Table S1: Detailed subset-level class-instance statistics of PV-Multi-Defect-main; Table S2: Detailed subset-level class-instance statistics of PVEL-AD (Part A); Table S3: Detailed subset-level class-instance statistics of PVEL-AD (Part B).
