# Supplementary material for: MSW-Mamba-Det: Multi-Scale Windowed State-Space Modeling for End-to-End Defect Detection in Photovoltaic Module Electroluminescence Images
Source: Sensors (Basel). 2026 Apr 23;26(9):2616. doi: 10.3390/s26092616 (PMC13165787; doi:10.3390/s26092616)
Supplement: Supplementary file 1 [file sensors-26-02616-s001.zip › sensors-4221074-supplementary.pdf]

Supplementary Materials

This supplementary file p rovides d etailed s ubset-level c lass-instance s tatistics f or t he two datasets used in this study. For both datasets, the reported counts correspond to the training, validation, and test subsets used in the main experiments.

S1. Detailed Split Statistics of PV-Multi-Defect-main

Table S1. Detailed subset-level class-instance statistics of PV-Multi-Defect-main.

Split	Images	Instances	hot_spot	scratch	no_electricity	black_border	broken_area
Train	773	2734	1392	960	124	188	70
Validation	166	626	338	209	31	33	15
Test	166	621	349	198	26	35	13
Total	1105	3981	2079	1367	181	256	98

S2. Detailed Split Statistics of PVEL-AD

Table S2. Detailed subset-level class-instance statistics of PVEL-AD (Part A).

Split	Images	Instances	black_core	corner	crack	finger	fragment	horizontal_dislocation
Train	3220	5863	718	24	886	2075	22	558
Validation	690	1293	156	8	207	455	4	120
Test	691	1324	161	4	208	469	4	120
Total	4601	8480	1035	36	1301	2999	30	798

Table S3. Detailed subset-level class-instance statistics of PVEL-AD (Part B).

Split	printing_error	scratch	short_circuit	star_crack	thick_line	vertical_dislocation
Train	335	35	344	91	679	96
Validation	64	7	74	22	155	21
Test	79	12	74	26	147	20
Total	478	54	492	139	981	137
